# PAVFCOS: The development of a core outcome set for pouch anal and vaginal fistula

**DOI:** 10.1111/codi.70184

**Published:** 2025-08-08

**Authors:** Lillian Reza, Lara Bapir, Nusrat Iqbal, Charlene Sackitey, Sally Hughes, Mina Babbar, Rali Marinova, Petya Marinova, Pearl Avery, Phillip Lung, Jonathan P. Segal, Pär Myrelid, Paolo Gionchetti, Bram Verstockt, Ailsa Hart, Sue K. Clark, Phil Tozer

**Affiliations:** ^1^ Robin Phillips Fistula Research Unit St Mark's Hospital, Imperial College London London UK; ^2^ West Middlesex University Hospital Isleworth UK; ^3^ IBD Unit St Mark's Hospital, Imperial College London London UK; ^4^ The Royal Melbourne Hospital Melbourne Victoria Australia; ^5^ The University of Melbourne Melbourne Medical School Melbourne Victoria Australia; ^6^ Department of Surgery in Linköping, and Department of Biomedical and Clinical Sciences Linköping University Linköping Sweden; ^7^ IBD Unit, Department of Medical and Surgical Sciences, S. Orsola‐Malpighi Hospital University of Bologna Bologna Italy; ^8^ Department of Gastroenterology and Hepatology University Hospitals Leuven Leuven Belgium; ^9^ St Mark's Hospital Imperial College London London UK

**Keywords:** core outcome set, ileoanal pouch, inflammatory bowel disease, patient‐reported outcomes

## Abstract

**Aims:**

The primary aim was to develop a patient‐centred core outcome set (COS) for interventional studies in patients with pouch anal and vaginal fistula (PAVF).

**Method:**

PAVFCOS was developed using the methodology outlined by the Core Outcome Measures in Effectiveness Trials (COMET). Systematic review and qualitative patient interviews produced a long list of candidate outcomes. An online Delphi exercise was conducted with stakeholders to prioritise and shortlist outcomes. A consensus meeting of patients and clinicians ratified the final core outcome set.

**Results:**

A systematic review of the literature and 14 qualitative patient interviews produced a long list of 99 outcomes. These were reduced to 46 outcomes and subjected to two rounds of a Delphi exercise with 70 participants, including patients (45%), colorectal surgeons (31%), radiologists (6%), gastroenterologists (11%) and pouch specialist nurses (7%). Thirty‐six outcomes were discussed in a consensus meeting of seven clinicians and seven patients. Seven core outcomes were selected for inclusion: global assessment of continence, pain related to fistula and surrounding area, impact on quality of life of fistula discharge, fistula healing (clinical and radiological), new fistula or abscess, need for rescue intervention (minor or major) and global quality‐of‐life assessment.

**Conclusion:**

PAVFCOS is the first to establish outcomes that are important to patients with pouch anal and vaginal fistula. PAVFCOS should be used in interventional studies to introduce much‐needed standardisation of outcome reporting in this challenging condition.

**What does this paper add to the literature?**

Studies in pouch fistula report varied outcomes of numerous interventions, often in small case series. Heterogeneous outcome reporting precludes meta‐analysis of an already limited evidence base. The use of PAVFCOS in future studies will improve the quality of the evidence and allow for comparisons between interventional approaches.

## INTRODUCTION

The ileoanal pouch is the gold standard restorative procedure for ulcerative colitis (UC) and familial adenomatous polyposis (FAP) syndrome [1]. Pouch anal and vaginal fistula (PAVF) has a prevalence of 5% and can cause debilitating symptoms, including perianal pain and discharge, affecting quality of life and pouch function [2, 3]. Fistulae may develop due to anastomotic leak at the pouch anal anastomosis, inflammatory bowel disease with or without clinical features of Crohn's disease, cryptoglandular disease and malignancy [4]. Despite advances in surgical practice, the failure rate of local reparative treatment remains approximately 50% since the inception of the ileoanal pouch [5, 6]. Existing literature primarily consists of single‐institution case series of interventional procedures for vaginal fistula [6] with variability in reparative techniques, follow‐up duration and outcome reporting [5, 6]. The reported outcomes are clinician‐driven and may not be relevant to patients. Outcomes reported by clinicians are not always concordant with those outcomes that are important to patients following ileoanal pouch surgery [7]. The patient perspective is increasingly becoming central to clinical research on the ileoanal pouch, reflecting a welcome shift towards integrating patient‐reported outcomes in inflammatory bowel disease (IBD) studies [8]. Patient‐centred and standardised outcome reporting is crucial to improve clinical management as it will allow comparisons between interventions and enable meta‐analysis of studies [6]. Core outcome sets (COS) have been developed to reduce heterogeneity in outcome reporting by establishing a minimum set of outcomes that should be measured and reported in all clinical studies of a particular health condition [9]. Disease‐specific COS have been developed for patients with Crohn's disease and cryptoglandular disease‐related fistula by our research team [10, 11].

Using methodology outlined by the Core Outcome Measures in Effectiveness Trials (COMET) initiative, we developed a disease‐specific, patient‐centred COS for use in all clinical studies of adult patients with pouch anal and vaginal fistula [12].

## METHOD

### Protocol registry

The PAVFCOS study protocol is registered on the COMET database and can be accessed at https://www.comet‐initiative.org/Studies/Details/1398. PAVFCOS has been reported using the COS‐STAndards for Reporting (COS‐STAR) statement [13].

### Scope

Patients may undergo surgical interventions, medical treatment or a combination. PAVFCOS includes outcomes that can be reported in all research studies of adult patients with pouch anal or vaginal fistula irrespective of treatment modality.

### Study management group

The study management group (SMG) included a gastroenterologist, a radiologist, two colorectal surgeons, two pouch specialist nurses and two patient representatives with personal experience of living with a pouch fistula. The clinicians involved in the SMG practice in a tertiary referral unit with a high‐volume experience of managing patients with pouch fistula and have methodological experience of developing disease‐specific core outcome sets (PT, AH, PL) [10, 11].

### Participants

Patients with a pouch anal or vaginal fistula in the 12 months preceding the study were included and recruited through clinics, social media and patient support groups. Clinicians who identified themselves as either having routine clinical exposure to patients with pouch fistula (>5 per year) or a research interest were included. Clinicians were recruited via social media posts and invitations through speciality associations.

### Identification of candidate outcomes

A systematic literature review and qualitative interviews were used to identify clinician and patient‐reported outcomes.

### Systematic review

The Preferred Reporting Items for Systematic Reviews and Meta‐Analyses (PRISMA) statement was used for the systematic review of the literature [14]. Embase, Medline and The Cochrane Library were searched for studies evaluating medical, surgical and combined interventions in adult patients with pouch anal or vaginal fistula, using a broad search strategy developed with an information specialist (Table S1).

The search was limited to full‐text articles of interventional studies published after the introduction of the pelvic pouch from January 1979 to March 2022. Retrospective and prospective studies reporting outcomes following intervention for fistula were included. Studies with subgroup analyses of patients with pouch fistula were included. Systematic reviews of the management of pouch fistula were included to extract reported outcomes, and their references were screened for additional studies. Studies were excluded if they were only available in abstract form, did not report on outcomes of pouch anal or vaginal fistula, included interventions other than medical or surgical treatment or were not in the English language.

Four independent reviewers (LR, LB, CS and NI) screened studies using the Covidence Systematic Review Software (Veritas Health Innovation, Melbourne, Australia; available at www. covidence.org). Any disagreement on study eligibility was resolved by discussion with recourse to senior authors (PT, SC and AH) if necessary. Two members of the study team (LR and LB) independently extracted data using Microsoft Excel (version 16.69.1).

### Patient interviews

Maximum variation purposive sampling was used for patient recruitment to ensure representation of patients at various stages of disease, pouch fistula aetiology, pouch fistula type (anal or vaginal) and ethnicity. The study management group identified four possible states of disease: (1) patients with a symptomatic fistula, post‐treatment; (2) patients with an asymptomatic fistula, post‐treatment; (3) patients with a symptomatic fistula, no treatment; and (4) patients with an asymptomatic fistula, no treatment.

Semi‐structured interviews were conducted using a predefined interview guide by a clinician–researcher trained in qualitative interview methodology (LR). The SMG developed the interview guide to limit interviewer bias and ensure patient perspectives were adequately captured (Supporting Information). Interviews were conducted using Zoom or telephone. Interviews were audio‐recorded, anonymised and transcribed verbatim. Two study team members performed a thematic analysis of the transcribed interviews. Thematic analysis was reviewed by a senior author (PT) and patient representatives from the SMG. Thematic analysis was conducted using the framework method with an inductive approach. Open coding was used to identify themes and categories. Transcribed interviews were coded, categorised and organised into a matrix using Microsoft Excel. Patient recruitment was concluded upon reaching thematic saturation, defined as the point at which no new themes emerged in three consecutive interviews.

### Outcome categorisation

The SMG removed outcomes that were duplicates, procedure‐specific, immeasurable or were unrelated to the assessment of treatment success. The outcomes were grouped according to a common theme and categorised by domain using the COMET taxonomy [9].

### Delphi exercise

Two rounds of Delphi surveys were conducted using DelphiManager, developed by the COMET Initiative. Definitions were agreed a priori to establish the consensus thresholds required for inclusion of outcomes for discussion in the final consensus meeting, summarised in Table 1.

**TABLE 1:** Thresholds for inclusion in final consensus meeting.

Consensus status	Definition	Description
Consensus in	>70% of the participants in each panel rating the outcome 7–9 OR < 70% of the clinicians rating the outcome 7–9 but a patient median rating of >7 AND 70% of the patients rating the outcome 7–9	Important outcome, for discussion in consensus meeting
Consensus out	<70% of the clinicians rating the outcome 7–9 AND a patient median rating of <7	Unimportant outcome, not for further discussion
No consensus	Anything else	Unclear importance, for further discussion in consensus meeting

Patient representatives on the SMG produced lay definitions to accompany each outcome (Table S6). A 9‐point Likert scale was used to score how important participants thought an outcome was in determining whether an intervention for a fistula had been successful. A score of 1–3 is ‘not important’, 4–6 is ‘fairly important’, and 7–9 is ‘very important’. Participants could propose additional outcomes at the end of Round 1. Additional outcomes were discussed by the SMG to ascertain whether these were truly new outcomes, were measurable and within the scope of this study prior to inclusion in Round 2. Following Round 1, outcomes that reached the consensus threshold for exclusion were removed. Outcomes with ‘no consensus’ were taken to Round 2 of Delphi for prioritisation. In Round 2, participants could see their rating alongside a median rating and a graphical representation of how each panel voted on that outcome. Participants could change their rating with this new perspective of how important an outcome was to other stakeholders.

### Consensus meeting

Participants were presented with outcomes that reached the consensus threshold for inclusion and those with no consensus. Participants considered whether the outcome was (1) an important measure of treatment success, (2) universally applicable to all interventional studies and (3) could be combined with another outcome.

Participants voted ‘Yes’ for inclusion or ‘No’ for exclusion of an outcome. Outcomes with more than 70% of the vote for inclusion were forwarded for inclusion to the COS. The patient vote percentage was also noted for each outcome to ensure outcomes highly prioritised by patients were never excluded without discussion. Outcomes that did not meet inclusion criteria as a stand‐alone outcome were considered with other outcomes to assess whether they could be combined. Combined outcomes were presented to participants for inclusion in the COS. The SMG reviewed the final COS to ensure adherence to protocol.

## RESULTS

### Patient interviews

Patient interviews included 14 patients (9 females) from a range of ages with vaginal [7] and anal [9] fistula, representing four states of disease (Table S3). Median interview duration was 60 minutes (range 33–74). Thematic analysis of transcribed interviews found 66 possible outcomes for inclusion, which were further grouped into 35 themes and mapped to domains using the COMET taxonomy [9]. Patient interviews generated 18 outcomes not found in the systematic review (Tables S4 and S6).

### Systematic review

The literature search identified 1962 studies (Figure S1). Of the 70 studies included for data extraction, 68 were interventional studies in the management of pouch fistula and two were systematic reviews [5, 15] (Table S2). Outcomes are reported in 56 studies using retrospective data [16, 17, 18, 19, 20, 21, 22, 23, 24, 25, 26, 27, 28, 29, 30, 31, 32, 33, 34, 35, 36, 37, 38, 39, 40, 41, 42, 43, 44, 45, 46, 47, 48, 49, 50, 51, 52, 53, 54, 55, 56, 57, 58, 59, 60, 61, 62, 63, 64, 65, 66, 67, 68, 69, 70] and 12 using a prospective database [71, 72, 73, 74, 75, 76, 77, 78, 79, 80, 81, 82]. Outcomes of medical intervention were reported in 12 studies [17, 22, 24, 27, 30, 33, 35, 50, 56, 61, 79, 80]. Systematic review generated 72 outcomes, which were grouped into 28 themes by the SMG and mapped to domains within the COMET taxonomy [9] (Tables S5 and S6). Systematic review added 10 outcomes to the longlist, which were not reported by patients in interviews. The SMG suggested the inclusion of ‘global quality‐of‐life assessment’ as an outcome to reflect the use of various quality‐of‐life tools in studies.

### Delphi exercise

The Delphi exercise was conducted between July and November 2022. In Round 1, 81 participants registered for the study, with 78 rating all 46 outcomes (96% completion rate). In Round 2, 74 participants rated all 20 outcomes (94% completion rate). The attrition rate between rounds was 5%. The demographics of the participants are detailed in Table S7.

**FIGURE 1:**
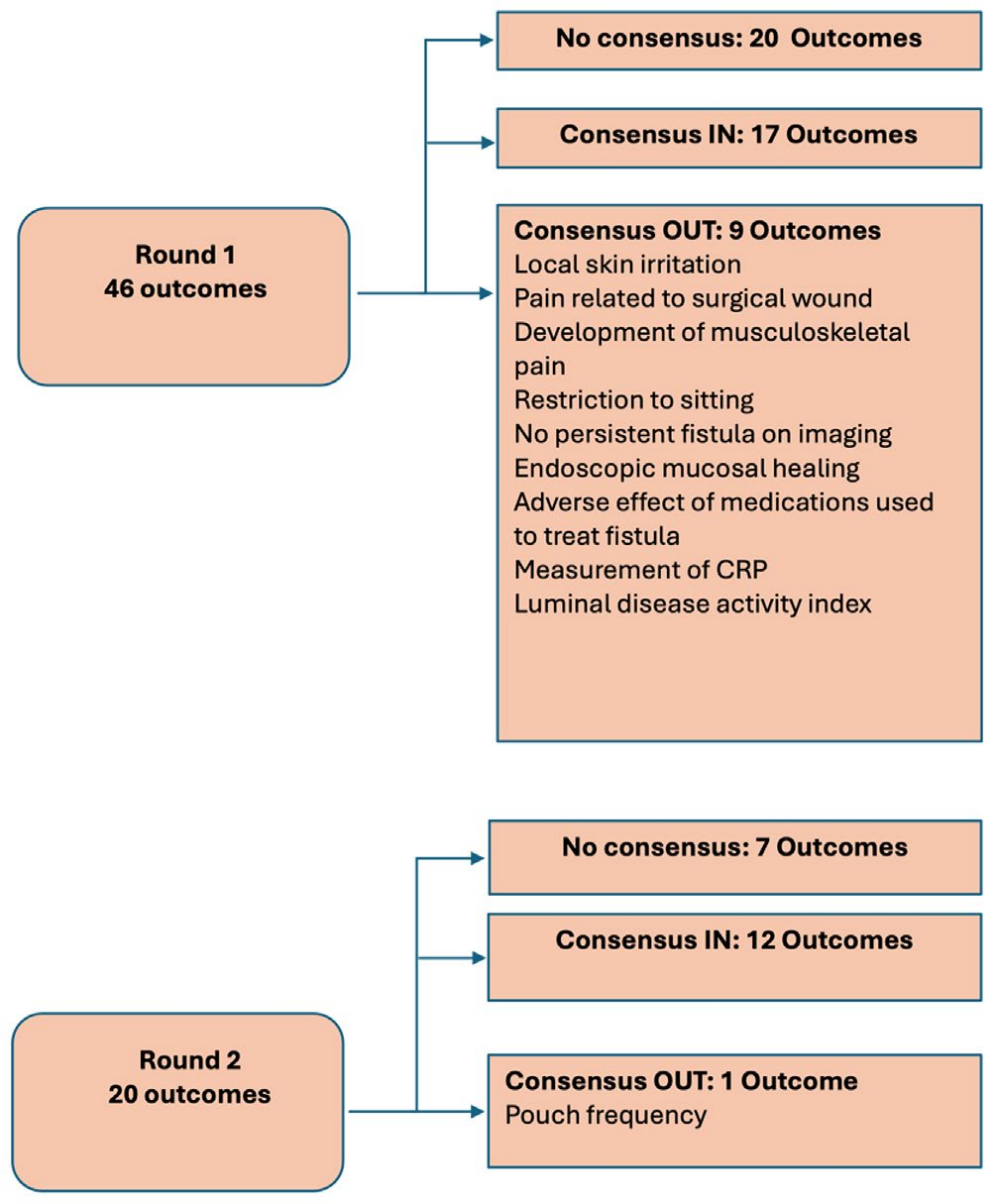
Summary of Delphi rounds.

Figure 1 displays the results of the ratings from each round. There were no new outcomes for addition to Round 2, and Round 3 of Delphi was not required.

### Consensus meeting

Thirty‐six outcomes were discussed for inclusion in the COS by seven patients and seven clinicians. The vote is summarised in Table S8. Of the seven outcomes with no consensus following Round 2 of Delphi, three outcomes were voted out (a change in the location of fistula opening, fatigue and need for a seton), and four were included as combined outcomes.

**FIGURE 2:**
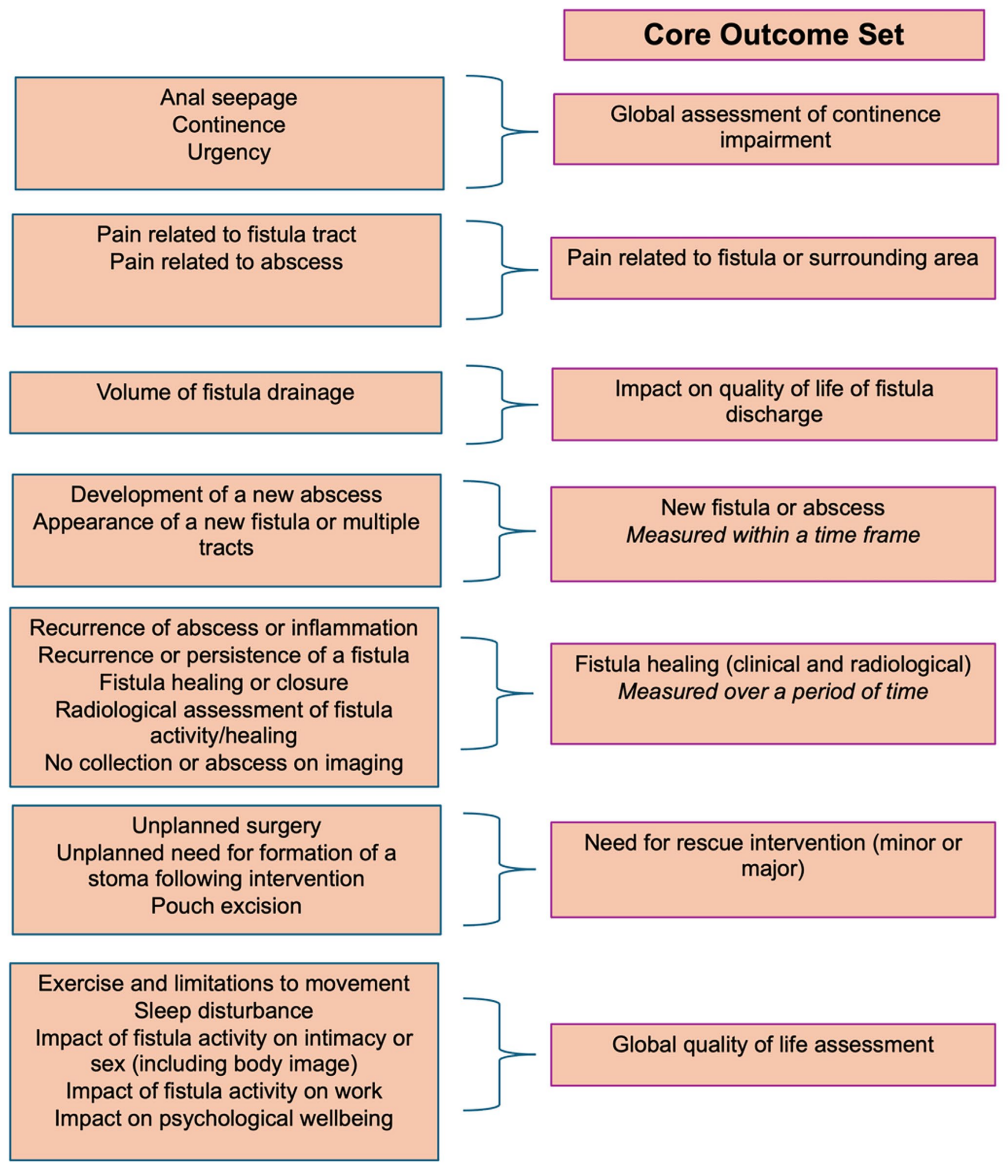
Summary of outcomes combined to generate core outcome set.

The final COS consists of seven outcomes (Figure 2). The consensus process for each outcome is outlined below.

#### Global assessment of continence impairment

‘Anal seepage’, ‘continence’ and ‘urgency’ were combined as ‘global assessment of continence impairment’ to represent the broad theme of incontinence.

#### Pain related to fistula or surrounding area

‘Anal pain’, ‘pain related to the fistula tract’ and ‘pain related to an abscess’ were combined as patients found it challenging to discriminate between these types of pain, and this construct represents pain following intervention or formation of a new abscess.

#### Impact on quality of life of fistula discharge

‘Volume of fistula discharge’ was an important outcome for patients due to its effect on quality of life rather than as an absolute volume measure. The outcome was thus reworded for measurement and evaluation in a COS.

#### Need for rescue intervention (minor or major)

‘Unplanned need for formation of a stoma’ following intervention and ‘pouch excision’ were grouped as ‘need for rescue intervention: minor or major’. Major interventions include ‘unplanned stoma formation’ and ‘pouch excision’. Minor intervention would include all other unplanned surgery performed shortly following reparative intervention for a fistula.

#### Global quality of life assessment

‘Psychological impact of needing treatment for a fistula’ and ‘fear and anxiety related to the impact on pouch function’ were not considered important for inclusion as stand‐alone outcomes. However, the group agreed that these outcomes were important to patients due to the considerable impact on quality of life. Patients strongly advocated for the inclusion of an outcome that measured psychological well‐being, as this is often overlooked in the clinical setting. A ‘global quality‐of‐life assessment’ should include measurement of ‘impact on psychological well‐being’, ‘impact of fistula activity on intimacy or sex’, ‘exercise and limitation to movement’ and ‘impact of fistula activity on work’.

#### Fistula healing (clinical or radiological)

‘Fistula healing or closure’, ‘recurrence or persistence of a fistula’ and ‘recurrence of abscess or inflammation’ were combined as they represent various clinical states of fistula healing. ‘Radiological assessment of healing’ and ‘no collection or abscess on imaging’ were included as these represent objective measures of fistula healing. Patients and clinicians highlighted the importance of establishing time points for the assessment of fistula healing on imaging to ensure standardised reporting in the literature.

#### New fistula or abscess

The group discussed combining ‘appearance of a new fistula or multiple fistula tracts’ and ‘development of a new abscess’ as the outcome ‘new fistula or abscess’. Consensus could not be reached regarding whether this outcome should be combined and retained as patients noted that a new fistula or abscess may not relate to the original fistula under assessment and may not be an accurate indication of treatment failure. Clinicians were concerned that exclusion of this outcome risks the under reporting of iatrogenic fistula from an intervention. The outcome did not reach the 70% threshold for inclusion in the COS. Of the patient group, 71% voted to discard the outcome. The consensus group suggested that the SMG further review this outcome. The SMG evaluated the outcome and concluded that a time frame should be defined for reporting a new fistula or abscess from index treatment to ensure these are related to the treatment episode under assessment.

## DISCUSSION

This study has established a COS to measure a minimum set of outcomes in all interventional studies of pouch anal and vaginal fistula. The COS will standardise outcome reporting and provide a holistic assessment of treatment success. The COS development exercise has added much‐needed nuance to definitions of relevant outcomes. In interviews, patients reported poor pouch function as an important measure of treatment success following fistula surgery. However, ‘pouch frequency’ was excluded in Round 2 of Delphi as it does not directly assess treatment success for a fistula. The COS established features related to continence, which were important to patients in the context of anal fistula surgery and its impact on sphincter function. A measurement tool for the global assessment of continence impairment in patients with an ileoanal pouch needs to be established. The Wexner incontinence score [83] and Vaizey incontinence score [84] have been used in the pouch fistula literature to assess incontinence although they are not validated for these patients. ‘Fistula healing’, ‘closure’ or ‘persistence’ are reported as primary outcomes in all studies in the systematic review but definitions for healing are missing, and only 2% of studies included Magnetic Resonance Imaging (MRI) for the assessment of fistula healing. This study highlights the importance of MRI for the assessment of fistula healing. MRI has been shown to demonstrate collection or persistent tracts that are predictive of long‐term failure of fistula healing and are often missed on clinical assessment alone [85, 86, 87]. The use of MRI cannot be mandated for the assessment of healing in all patients due to the implications on finite resources and the lack of accessibility to MRI in some clinical systems. However, MRI should be used with a low threshold to assess anal fistula suspected to be complex on clinical assessment [88, 89]. A strength of this study is the inclusion of patients in the study management group and all phases of COS development. Patient interviews and the Delphi survey included more female patients reflecting the literature which has a higher preponderance of vaginal fistula [6]. All disease states and fistula aetiology were represented by the participants. All stakeholders involved in the care of patients with pouch fistula were represented, including surgeons, gastroenterologists, radiologists and clinical nurse specialists of the ileoanal pouch. PAVFCOS has established the need for a disease‐specific quality‐of‐life scale, which will assess the impact of pouch fistula on patients' psychological well‐being, daily life, physical activity and intimacy. The next steps are the development of a core measurement set to identify instruments for use in the assessment of the core outcomes established in PAVFCOS as well as a disease‐specific scale to assess quality of life [90].

There are limitations of the COS that should be considered. The COS possibly has limited geographical representation as it was conducted in English. This may have limited participation from non‐English‐speaking patients and clinicians. Patients and clinicians were from the United Kingdom, Europe, North America and Australia, but only clinicians represented China, Turkey and Malaysia. Most patients were Caucasian, female and 57% were being treated at a tertiary referral centre, representing the complexity of the disease. Extreme patient experiences may bias outcome generation and prioritisation, but this is unlikely to have reduced the utility of the COS. Patients with pouch fistula are at a higher risk of pouch failure, demonstrating challenges of treating this condition [91]. This also affects the representation of clinicians involved in the study, with the majority practising in tertiary centres (97%), reflecting the highly specialised nature of disease management. Researchers in underrepresented regions may consider adopting the COS as a foundation for outcome reporting and plan for local validation or adaptation to ensure relevance to their populations.

A core outcome measurement set (COMS) study is currently underway to support the implementation of the COS. This study involves clinicians with expertise in pouch fistula care and patients with lived experience. It aims to identify and recommend validated instruments for measuring each core outcome, enhancing the COS's utility and facilitating its adoption in research. Finally, PAVFCOS has the potential to inform not only research but also clinical care. The COS could be adapted into a practical tool to support structured patient assessments in clinic, ensuring that both physical and quality‐of‐life outcomes are considered during decision‐making, enhancing patient‐centred care.

## CONCLUSION

PAVFCOS is the first step towards harmonising standards reported in the literature for patients with pouch fistula. Future clinical studies should include this COS in outcome reporting to improve the quality of research and strengthen the evidence base for the management of this challenging disease.

## AUTHOR CONTRIBUTIONS

**Lillian Reza:** Conceptualization; investigation; methodology; software; data curation; formal analysis; project administration; visualization; validation; writing – original draft. **Lara Bapir:** Software; data curation; investigation. **Nusrat Iqbal:** Software; data curation; investigation. **Charlene Sackitey:** Software; data curation; investigation. **Sally Hughes:** Validation; investigation. **Mina Babbar:** Validation; investigation. **Rali Marinova:** Validation; investigation; writing – review and editing. **Petya Marinova:** Validation; investigation; writing – review and editing. **Pearl Avery:** Validation; investigation; writing – review and editing. **Phillip Lung:** Validation; writing – review and editing; investigation. **Jonathan P. Segal:** Investigation; validation; writing – review and editing. **Pär Myrelid:** Investigation; validation; writing – review and editing. **Paolo Gionchetti:** Investigation; validation; writing – review and editing. **Bram Verstockt:** Investigation; validation; writing – review and editing. **Ailsa Hart:** Supervision; methodology; investigation; validation; writing – review and editing; resources. **Sue K. Clark:** Methodology; validation; investigation; writing – review and editing; supervision; resources. **Phil Tozer:** Conceptualization; methodology; validation; funding acquisition; investigation; visualization; writing – review and editing; supervision; resources.

## FUNDING INFORMATION

PAVFCOS was funded by the Robin Phillips Fistula Research Unit at St Mark's Hospital and Academic Institute.

## CONFLICT OF INTEREST STATEMENT

No conflict of interest to disclose.

## ETHICS STATEMENT

The study was granted ethics approval from the North of Scotland Research Ethics Service (REC reference 20/NS/0120).

## PATIENT CONSENT

Written consent was taken from patients for qualitative interviews. Electronic consent was established from all participants for the Delphi consensus exercise.

## STUDY REGISTRATION

The PAVFCOS study protocol was registered and published online in the Core Outcome Measures in Effectiveness Trials (COMET) database. This can be accessed at https://www.comet‐initiative.org/Studies/Details/1398. The conception and dissemination of the study were supported by the Ileostomy and Internal Pouch Association and the Red Lion Pouch Support Group at St Mark's Hospital.

## Supporting information


**Table S1.** Systematic review search strategy.
**Table S2.** Characteristics of included studies from systematic review.
**Table S3.** Demographics of patients interviewed.
**Table S4.** Verbatim patient‐reported outcomes from qualitative interviews.
**Table S5.** Outcomes extracted from systematic review.
**Table S6.** Outcomes included in the Delphi exercise with lay definitions.
**Table S7.** Delphi participant demographics.
**Table S8.** Consensus meeting voting results.
**Figure S1.** PRISMA for systematic review of outcomes in the management of pouch anal and vaginal fistula.

Data S1.

## Data Availability

The data that supports the findings of this study are available in the supplementary material of this article.

## Appendix

Collaborators: The authors thank the members of the PAVFCOS expert collaborators and patient panel for their contributions to the Delphi rounds and consensus meeting: Adam Haycock, Alison Corr, Andrew Plumb, Baljit Singh, Caroline Wilson, Carolynne Vaizey, Charles Knowles, Chris Lamb, David Burling, David Proud, Elaine Hui Been Ng, Esther Franklin, Giacomo Calini, Gianluca Pellino, Gordon Buchanan, Helen Jukes, Janindra Warusavitarne, Jasper Stijns, Joanna Skelton, Jonathan Weighill, Kate Williams, Katie Adams, Kevin Monahan, Laura Hancock, Lisa Massey, Michele Marshall, Nurulamin Noor, Piotr Eder, Rajapandian Ilangovan, Ripple Man, Robyn Moore, Samantha Evans, Samuel Adegbola, Sara Chatting, Semra Demirli Atici, Sezai Leventoglu, Steve Burnett, Steven D Wexner, Valerio Celentano, Wah Yang, Zarah Perry‐Woodford, Tricia Levasseur. [Correction added on 27 November 2025, after first online publication: Tricia Levasseur’s name has been added to the collaborator’s list in this version.].
