# Supplementary material for: PAVFCOS: The development of a core outcome set for pouch anal and vaginal fistula
Source: Colorectal Dis. 2025 Aug 8;27(8):e70184. doi: 10.1111/codi.70184 (PMC12334349; doi:10.1111/codi.70184)
Supplement: Supplementary file 1 — Table S1. Systematic review search strategy. Table S2. Characteristics of included studies from systematic review. Table S3. Demographics of patients interviewed. Table S4. Verbatim patient‐reported outcomes from qualitative interviews. Table S5. Outcomes extracted from systematic review. Table S6. Outcomes included in the Delphi exercise with lay definitions. Table S7. Delphi participant demographics. Table S8. Consensus meeting voting results. Figure S1. PRISMA for systematic review of outcomes in the management of pouch anal and vaginal fistula. [file CODI-27-e70184-s002.docx]

**Supplementary**

**Supplementary Table 1: Systematic search**

| \| 1 \| exp Rectal Fistula/ or exp Vaginal Fistula/ or exp Rectovaginal Fistula/ or exp Vesicovaginal Fistula/ or "PVF".ti,ab,kw,kf. \| 9540 \| \| --- \| --- \| --- \| \| 2 \| (anal or rectal or perine* or "peri-ne*" or vagina*).ti,ab,kw,kf. \| 252669 \| \| 3 \| Fistula/ or (fistula* or tract* or connect* or communicat*).ti,ab,kw,kf. \| 1183603 \| \| 4 \| 2 and 3 \| 27650 \| \| 5 \| 1 or 4 \| 32920 \| \| 6 \| ("ileal-pouch" or "ileal pouch" or "ileum-pouch" or "ileum pouch" or "ileo-anal" or "ileo anal" or "IAPP" or "IPAA" or reservoir* or pouch* or "j-pouch*" or "s-pouch*" or "w-pouch*" or "internal pouch" or "internal-pouch*" or (restorative adj3 proctocolectom*)).ti,ab,kw,kf. \| 83200 \| \| 7 \| Colonic Pouches/ or Anastomosis, Surgical/ or Proctocolectomy, Restorative/ \| 34095 \| \| 8 \| 6 or 7 \| 113231 \| \| 9 \| 5 and 8 \| 1481 \| \| 10 \| Child/ or Child, Preschool/ or Infant/ or Infant, Newborn/ or Infant, Low Birth Weight/ or Infant, Small for Gestational Age/ or Infant, Very Low Birth Weight/ or Infant, Extremely Low Birth Weight/ or Infant, Postmature/ or Infant, Premature/ or Infant, Extremely Premature/ or Pediatrics/ or Adolescent/ \| 3578965 \| \| 11 \| (p?ediatric* or child* or baby or babies or infant* or "neo nat*" or neo-nat* or neonat* or embryo* or newborn or adolesc*).ti,ab,kw,kf. \| 2598086 \| \| 12 \| 10 or 11 \| 4551318 \| \| 13 \| 9 not 12 \| 1024 \| \| 14 \| limit 13 to human \| 846 \| \| 15 \| limit 14 to yr="1978-current" \| 838 \| \| 16 \| remove duplicates from 15 \| 837 \| | **837** |
| --- | --- | --- | --- | --- | --- | --- | --- | --- | --- | --- | --- | --- | --- | --- | --- | --- | --- | --- | --- | --- | --- | --- | --- | --- | --- | --- | --- | --- | --- | --- | --- | --- | --- | --- | --- | --- | --- | --- | --- | --- | --- | --- | --- | --- | --- | --- | --- | --- | --- |
| \| 1 \| exp rectum fistula/ or exp anus fistula/ or exp rectovaginal fistula/ or exp cystovaginal fistula/ or "PVF".ti,ab,kw. \| 13871 \|  \| \| --- \| --- \| --- \| --- \| \| 2 \| (anal or rectal or perine* or "peri-ne*" or vagina*).ti,ab,kw. \| 352127 \|  \| \| 3 \| fistula/ or (fistula* or tract* or connect* or communicat*).ti,ab,kw. \| 1486638 \|  \| \| 4 \| 2 and 3 \| 42811 \|  \| \| 5 \| 1 or 4 \| 50008 \|  \| \| 6 \| ("ileal-pouch" or "ileal pouch" or "ileum-pouch" or "ileum pouch" or "ileo-anal" or "ileo anal" or "IAPP" or "IPAA" or reservoir* or pouch* or "j-pouch*" or "s-pouch*" or "w-pouch*" or "internal pouch" or "internal-pouch*" or (restorative adj3 proctocolectom*)).ti,ab,kw. \| 101733 \|  \| \| 7 \| ileal pouch-anal anastomosis/ or ileoanal anastomosis/ or ileorectal anastomosis/ or rectum anastomosis/ or ileum pouch/ or protocolectomy/ \| 6078 \|  \| \| 8 \| 6 or 7 \| 104238 \|  \| \| 9 \| 5 and 8 \| 2040 \|  \| \| 10 \| child/ or preschool child/ or infant/ or newborn/ or low birth weight/ or small for date infant/ or very low birth weight/ or extremely low birth weight/ or postmaturity/ or prematurity/ or pediatrics/ or adolescent/ \| 3371678 \|  \| \| 11 \| (p?ediatric* or child* or baby or babies or infant* or "neo nat*" or neo-nat* or neonat* or embryo* or newborn or adolesc*).ti,ab,kw. \| 3120759 \|  \| \| 12 \| 10 or 11 \| 4507519 \|  \| \| 13 \| 9 not 12 \| 1562 \|  \| \| 14 \| limit 13 to human \| 1310 \|  \| \| 15 \| limit 14 to yr="1978-current" \| 1308 \|  \| \| 16 \| remove duplicates from 15 \| 1302 \|  \| | **1302** |
| #1 ([mh "Rectal Fistula"] OR [mh "Vaginal Fistula"] OR [mh "Rectovaginal Fistula"]) OR "PVF":ti,ab,kw 230  #2 (anal OR rectal OR perine* OR "peri-ne*" OR vagina*):ti,ab,kw 34694  #3 [mh ^Fistula] OR (fistula* OR tract* OR connect* OR communicat*):ti,ab,kw 79324  #4 #2 AND #3 3303  #5 #1 OR #4 3350  #6 ("ileal-pouch" OR "ileal pouch" OR "ileum-pouch" OR "ileum pouch" OR "ileo-anal" OR "ileo anal" OR "IAPP" OR "IPAA" OR reservoir* OR pouch* OR "j-pouch*" OR "s-pouch*" OR "w-pouch*" OR "internal pouch" OR "internal-pouch*" OR (restorative NEAR3 proctocolectom*)):ti,ab,kw 4540  #7 ([mh ^"Colonic Pouches"] OR [mh ^"Anastomosis, Surgical"] OR [mh ^"Proctocolectomy, Restorative"]) 828  #8 #6 OR #7 5189  #9 #5 AND #8 68  #10 ([mh ^Child] OR [mh ^"Child, Preschool"] OR [mh ^Infant] OR [mh ^"Infant, Newborn"] OR [mh ^"Infant, Low Birth Weight"] OR [mh ^"Infant, Small for Gestational Age"] OR [mh ^"Infant, Very Low Birth Weight"] OR [mh ^"Infant, Extremely Low Birth Weight"] OR [mh ^"Infant, Postmature"] OR [mh ^"Infant, Premature"] OR [mh ^"Infant, Extremely Premature"] OR [mh ^Pediatrics] OR [mh ^Adolescent]) 146352  #11 (p?ediatric* or child* or baby or babies or infant* or "neo nat*" or neo-nat* or neonat* or embryo* or newborn or adolesc*):ti,ab,kw 290811  #12 #10 OR #11 290811  #13 #9 NOT #12 with Cochrane Library publication date Between Jan 1978 and March 2022 58 | **CDSR - 3**  **CENTRAL - 55** |

**Supplementary Table 2: Characteristics of included studies from systematic review of management of pouch anal and vaginal fistula**

Extracted data includes: first author, journal, year of publication, study type, total number of patients, number of anal fistula, number of vaginal fistula, aetiological diagnosis of fistula (where reported), outcomes measured, outcome definitions, measurement instrument used for outcomes and timing of outcome assessment.

| First author | Journal | Year of publication | Study type | Number of fistulas | PAF | PVF | Fistula aetiology | Intervention |
| --- | --- | --- | --- | --- | --- | --- | --- | --- |
| Araki et al | Inter J Colorectal disease | 2017 | Retrospective | 3 |  | 3 | Anastomotic leak | Martius graft |
| Araki et al | Surgery Today | 2014 | Retrospective | 2 | 2 | 0 | Crohns’ disease | Redo IPAA, Biologic |
| Burke et al | British J of Surgery | 2001 | Retrospective | 14 |  | 14 | Anastomotic leak | Transvaginal repair |
| Cohen et al | World J Surgery | 1998 | Retrospective | 12 |  | 12 | Anastomotic leak | Redo IPAA |
| Dehni et al | British Journal of Surgery | 2005 | Retrospective | 17 | 6 | 11 | Anastomotic leak | Transanal or abdominoperineal pouch advancement |
| Devesa et al | Tech Coloproctol | 2007 | Retrospective | 11 |  | 11 | Crohn’s disease | Fistulectomy and sphincteroplasty, Redo IPAA |
| Faucheron et al | Dis Colon Rectum | 2001 | Retrospective | 2 | 1 | 1 | Anastomotic leak | Endoanal ileal advancement flap technique |
| Ferrante et al | Inflammatory bowel disease | 2010 | Retrospective | 7 | 2 | 5 | Non-Crohn’s IBD | Infliximab |
| Furst et al | Inter J Colorectal disease | 2008 | Retrospective | 12 |  | 1 | Crohn’s disease | Gracilis muscle transposition |
| Gaertner et al | Tech coloproctol | 2014 | Retrospective | 25 | 12 | 7 | Cryptoglandular, Crohn’s or Anastomotic leak | Various- Cutting seton, GMT, Laying open, Martius flap, Collagen plug insertion, Ileal advancement flap, seton and anti TNF therapy, transperineal repair, fibrin glue |
| Gajsek et al | Dis Colon Rectum | 2011 | Prospective | 11 |  | 11 | No reported | Button plug |
| Gilshtein et al | Tech Coloproctol | 2020 | Retrospective | 9 |  | 2 | Crohn’s disease | Redo gracilis muscle transposition |
| Gonsalves et al | Dis Colon Rectum | 2009 | Prospective | 7 |  | 7 | Crohn’s disease | Button plug |
| Gorenstein et al | Diseases of colon and rectum | 1988 | Retrospective | 2 |  | 2 | Not reported | Gracilis muscle transposition |
| Gorfine et al | Diseases of colon and rectum | 2003 | Prospective | 51 |  | 7 | Not reported | Transanal or abdominoperineal pouch advancement |
| Gregory et al | Inflammatory bowel disease | 2019 | Retrospective | 26 | 17 | 9 | Non-Crohn’s IBD | Vedolizumab |
| Groom et al | British Journal of Surgery | 1993 | Retrospective | 22 |  | 22 | Non-Crohn’s IBD and Anastomotic leak | Transperineal and transvaginal repair |
| Halverson et al | Surgery | 2001 | Retrospective | 5 |  | 5 | Not reported | Ileal pouch advancement and Redo IPAA |
| Haveran et al | DIsease of Colon and Rectum | 2011 | Retrospective | 15 | 11 | 3 | Non-Crohn’s IBD | Infliximab and azathioprine |
| Heriot et al | Diseases of colon and rectum | 2005 | Retrospective | 68 |  | 68 | Anastomotic leak, Cryptoglandular disease | Local repair and abdominoperineal repair |
| Heuschen et al | British Journal of Surgery | 2002 | Retrospective | 107 |  | 15 | Not reported | Local repair and abdominoperineal repair |
| Huang et al | Gastroenterology | 2003 | Retrospective | 2 |  | 2 | Crohn’s disease | Infliximab |
| Hulten | The Netherlands Journal of medicine | 1994 | Retrospective | 5 | 3 | 2 | Not reported | Local repair and Redo IPAA |
| Johnson et al | Diseases of colon and rectum | 2005 | Retrospective of a prospective database | 29 |  | 29 | Not reported | Local repairs included fibrin glue injection, simple suture closure, transvaginal or transanal flap advancement and circumferential mo- bilization of the pouch through the anus with ad- vancement and reanastomosis below the fistula |
| Kato et al | Journal of Japanese society of gastroenterology | 2019 | Retrospective | 1 |  | 1 | Non-Crohn’s IBD | Adalimumab |
| Keighley et al | British Journal of Surgery | 1993 | Retrospective | 27 | 3 | 10 | Non-Crohn’s IBD and Anastomotic leak | Cutting seton, defunctioning ileostomy, transvaginal repair |
| Kjaer et al | Scandinavian journal of surgery | 2016 | Retrospective | 48 | 29 | 19 | Non-Crohn’s IBD and Anastomotic | Local repair and abdominoperineal repair |
| Korsgen et al | British Journal of Surgery | 1996 | Retrospective | 5 |  | 5 | Not reported | Seton fistulotomy with sphincter reconstruction |
| Korsun et al | Techniques in coloproctology | 2018 | Retrospective | 32 |  | 3 | Crohn’s disease | Gracilis muscle transposition |
| Lee et al | Diseases of Colon and Rectum | 1997 | Retrospective | 25 |  | 25 | Cryptoglandular, Crohn’s disease or Anastomotic leak | Local repair and Redo IPAA |
| Loungnarath et al | Diseases of colon and rectum | 2004 | Prospective | 39 |  | 4 | Not reported | Fibrin glue |
| Maclean et al | Diseases of colon and rectum | 2002 | Retrospective of a prospective database | 23 | 2 | 21 | Non-Crohn’s IBD and Anastomotic | Local repair and abdominoperineal repair |
| Mallick et al | Diseases of colon and rectum | 2014 | Retrospective of a prospective database | 102 |  | 102 | Crohn’s disease or Anastomotic leak | Local repair and abdominoperineal repair |
| Maslekar et al | Techniques in Coloproctology | 2012 | Systematic review |  |  |  |  | Local repair and abdominoperineal repair |
| Machin et al | Colorectal disease | 2020 | Systematic review |  |  |  |  | Local repair and abdominoperineal repair |
| Mathis et al | Annals of surgery | 2009 | Retrospective | 23 | 5 | 7 | Non-Crohn’s IBD and Anastomotic | Partial reconstructon of the pouch vs complete reconstruction |
| Mennigen et al | Langenbeck's Archives of Surgery | 2012 | Retrospective | 12 | 1 | 1 | Anastomotic leak | Local repair |
| Muhlmann et al | Australasian Journal of Surgery | 2010 | Retrospective | 70 |  | 1 | Not reported | Fistula plug |
| O'Kelly et al | British Journal of Surgery | 1994 | Retrospective | 6 |  | 6 | Anastomotic leak | Endovaginal flap advancement |
| Ogunbiyi et al | Diseases of colon and rectum | 1997 | Retrospective | 10 | 4 | 6 | Anastomotic leak | Defunctioning loop ileostomy |
| Ozuner et al | Diseases of colon and rectum | 1997 | Retrospective | 51 | 16 | 24 | Not reported | Local and abdominoperineal repair |
| Panis et al | Lancet | 1996 | Retrospective | 4 | 3 | 1 | Not reported | Gracilis muscle transposition with defunctioning |
| Peygrene et al | International journal of Colorectal disease | 1999 | Retrospective | 4 | 3 | 1 | Not reported | Gracilis muscle transposition with defunctioning |
| Raval at al | Annals of surgery | 2007 | Retrospective | 141 | 8 | 18 | Crohn’s disease, anastomotic leak | Pouch reconstruction, seton, local repair |
| Ricart et al | Gastroenterology | 1999 | Retrospective | 4 |  | 3 | Crohn’s disease | Infliximab |
| Rius et al | Eur J Surg | 2000 | Retrospective | 1 |  | 1 | Not reported | Gracilis muscle transposition |
| Rottoli et al | Updates in Surgery | 2018 | Retrospective of a prospective database | 25 |  | 7 | Not reported | Gracilis muscle transposition with defunctioning |
| Rottoli et al | Diseases of colon and rectum | 2018 | Retrospective | 13 |  | 12 | Not reported | Redo IPAA or pouch revision |
| Ryoo et al | World J Gastro | 2014 | Retrospective | 9 |  | 5 | Not reported | Transanal advancement flap |
| Sagar et al | Diseases of colon and rectum | 1996 | Retrospective | 15 | 4 | 3 | Anastomotic leak, Crohn’s disease | Local repair |
| Sagar et al | Colorectal disease | 2014 | Retrospective | 11 |  | 11 | Anastomotic leak | Transvaginal repair |
| Saigusa et al | International journal of Colorectal disease | 2015 | Retrospective | 1 |  | 1 | Cryptoglandular | Local repair |
| Sapci et al | Diseases of colon and rectum | 2019 | Retrospective of a prospective database | 70 |  | 70 | Crohn’s disease, anastomotic leak | Redo or Ileal pouch advancement |
| Shah et al | Diseases of colon and rectum | 2003 | Retrospective | 60 |  | 60 | Crohn’s disease | Local repair and Redo IPAA |
| Shen et al | Alimentary pharmacology and therapeutics | 2008 | Prospective | 5 |  | 5 | Crohn’s disease | Adalimumab |
| Sivathondan et al | Colorectal disease | 2020 | Retrospective | 23 |  | 23 | Crohn’s disease | Infliximab or adalumimab |
| Smith et al | American surgeon | 2007 | Retrospective | 1 |  | 1 | Anastomotic leak | Collagen permacol patch repair |
| Sofo et al | Techniques in coloproctology | 2018 | Retrospective | 1 |  | 1 | Not reported | Fasciocutaneous lotus petal flap |
| Thekkinkattil et al | Colorectal disease | 2009 | Retrospective | 45 |  | 3 | Not reported | Surgisis Fistula plug |
| Tran et al | Diseases of colon and rectum | 1999 | Retrospective | 4 |  | 4 | Not reported | Transposition of rectus abdominis with defunctioning ileostomy |
| Troja et al | Journal of visceral surgery | 2013 | Retrospective | 4 |  | 4 | Not reported | Gracilis muscle transposition |
| Tsujinaka et al | Journal of American college of surgeons | 2006 | Retrospective | 23 |  | 23 | Non- Crohn’s IBD, Crohn’s disease, anastomotic leak related | Local repair and Redo |
| Vallicelli et al | American surgeon | 2017 | Retrospective | 1 |  | 1 | Not reported | Redo gracilis muscle transposition |
| Viazis et al | Journal of Chrohn's and colitis | 2013 | Prospective | 3 | 2 |  | Non- Crohn’s IBD | Infliximab |
| Viscido et al | European Review for Medical and Pharmacological Sciences | 2004 | Retrospective | 8 | 3 | 4 | Non- Crohn’s IBD | Infliximab |
| Wainstein et al | Colorectal disease | 2018 | Prospective | 9 |  | 2 | Crohn’s disease | Local repair and Redo IPAA |
| Wexner et al | Diseases of colon and rectum | 1989 | Retrospective | 21 |  | 21 | Crohn’s disease, anastomotic leak | Mucosal advancement flap and infiltration with adepose derived mesenchymal stem cells |
| Yeung et al | Colorectal disease | 2008 | Retrospective | 40 |  | 4 | Not reported | Fibrin glue |
| Zinicola et al | British Journal of Surgery | 2003 | Retrospective | 38 |  | 38 | Not reported | Local repair and Redo IPAA |
| Zmora et al | Diseases of colon and rectum | 2006 | Retrospective | 1 |  | 1 | Not reported | Gracilis muscle transposition |

**Supplementary Table 3: Demographics of patients interviewed**

| Female | 9 |
| --- | --- |
| Male | 5 |
| **Age** | |
| 20-30 | 3 |
| 31-40 | 3 |
| 41-50 | 3 |
| 51-60 | 3 |
| 61-70 | 1 |
| **Ethnicity** | |
| Caucasian | 11 |
| Asian | 2 |
| Arab | 1 |
| **Fistula aetiology** | |
| Anastomotic leak related fistula | 5 |
| Inflammatory Type A- Crohn’s | 3 |
| Inflammatory Type B- Non-Crohn’s | 3 |
| Cryptoglandular disease | 2 |
| **Fistula type** | |
| Vaginal fistula | 4 |
| Anal fistula | 6 |
| Both | 3 |
| **Treatment status** | |
| Defunctioned | 1 |
| Biologics | 4 |
| Active fistula, no treatment | 5 |
| Active fistula, post treatment | 3 |
| Non-active fistula- post treatment | 4 |
| Non active fistula, no treatment | 1 |

**Supplementary Table 4: Verbatim patient reported outcomes from qualitative interviews mapped to broad outcome**

| Outcome theme | Patient reported outcomes |
| --- | --- |
| Fistula healing | 1. *Fistula is closed because no discharge. 2. *No opening on the outside. |
| Pouch frequency | 1. Deterioration in pouch function following surgery or pouch function so poor that it needs medical treatment. 2. *Need to use loperamide regularly so discharge through fistula is less 3. *Need to use codeine for discharge 4. Increase in the need to empty pouch. 5. Increase in the need to empty pouch during the night. |
| Urgency | 1. *Feeling like I need to empty pouch |
| Anal seepage | 1. *Seepage might get worse after operation for fistula |
| Continence | 1. *Preserve continence as I am now, and not make things worse 2. *I am soiling my clothes, and I need to wear pads anyway. I don’t want that to get worse |
| Volume of discharge | 1. Frequency of discharge is either continuous or intermittent. 2. *I know it hasn’t worked because discharge is worse than before 3. *No discharge to me means success. 4. *I think if the opening of the fistula is widened with surgery then this may make discharge much worse. This to me is a failure of treatment 5. Change in discharge type: faeces, pus, fistula leakage, air. *I only had liquid come through for a while, and then I noticed it was actually faeces so things had changed 6. Vaginal fistula discharge. *I had discharge from my vagina and that made me think I have another fistula. 7. Pus drainage instead of usual pouch contents is a sign of treatment failure. *I know it’s infection if the colour changes. Fistula has flared up. 8. *If I could not control leakage of faeces through fistula after the operation |
| Pain related to fistula tract | 1. *If pain around my fistula was better than I know treatment has worked 2. *Pain from fistula because of inflammation or if there’s an increase in drainage then I know fistula is active again 3. Dependent on analgesia due to worsening pain from fistula tract. 4. *Theres always a pressure when I am trying to empty my pouch when the fistula is active and inflammed |
| Pain related to an abscess | 1. *Pain from another abscess |
| Anal pain | 1. *Back passage pain. 2. *Sphincter spasms after an operation. |
| Local skin irritation | 1. *Skin irritation and *chaffing from discharge and fistula. |
| Pad usage | 1. Number of pads used increases after operation. *I always need to use more pads after an operation because of discharge |
| Recurrence or persistence of a fistula | 1. Recurrence of same fistula following operation. |
| Recurrence of abscess or inflammation | 1. Recurrent infection or abscess. |
| Development of a new abscess | 1. New abscess following operation. 2. The need to use antibiotics for new abscess or collection following operation. *I usually get antibiotics from GP if theres a flare up and it helps |
| Appearance of a new or multiple fistula tracts | 1. Development of a new tract following operation 2. Multiple tracts appear following surgery for one fistula tract. *I don’t think surgery has worked if there are more fistulas |
| Unplanned surgery | 1. Need for another operation to treat infection soon after the last operation. *If I had to have another operation for infection then I know that the first operation hasn’t worked |
| Unplanned need for formation of a stoma following intervention | 1. Treatment has failed if there is a need for a stoma following a procedure due to worsening of symptoms. *I do not want a stoma. My experience with my first stoma was very bad and I had to have that as an emergency. |
| Feeling unwell or malaise | 1. *Feeling generally unwell. This usually happens when discharge increases or there’s an infection near the fistula. 2. *Malaise, usually because of an abscess. |
| Pain related to surgical wound | 1. Post operative wound pain at the site of surgery. *I expected a bit of pain after surgery 2. Pain from packing of abscess cavity or wound. *I had to have the would packed and that was terrible. |
| Wound healing | 1. Bleeding at site of operation. 2. Non-healing site of operation. 3. *Wound size is too large. It took a long time to heal. Had to take time off work |
| Pouch excision | 1. Pouch excision following surgery to heal fistula. 2. *Might need to remove pouch if discharge is worse 3. *Maintain pouch as is following operation. |
| Fear and anxiety related to impact on pouch function | 1. Fear and anxiety related to worsening pouch function as a result of intervention. 2. Worsening pouch function can impact fistula drainage. Anxiety related to needing to control pouch function in order to reduce impact on fistula activity. 3. Fear of loss of pouch function. |
| Fatigue | 1. *Feeling tired, *drained, *weak as a result of fistula activity. 2. Fatigue from being *unable to sleep at night due to fistula activity.*I have to wake up to empty pouch at night but its worse if fistula is discharging a lot. I am too tired during the day. |
| Worried that exercise or sex might worsen symptoms | 1. Anxiety and worry that walking, running, standing or laying in a certain position may worsen fistula symptoms. *I try not to sit on it when its inflamed. *I can’t do the things I used 2. Avoiding sex as concerned that it might contribute to worsening symptoms of the fistula. *I would rather not because it’s in an awkward position. *It’s just not comfortable and discharge makes everything sore |
| Body image | 1. Impact on self-confidence or self perception from fistula symptoms. 2. Impact on self image from stoma or surgery needed for fistula. |
| Psychological impact of needing treatment for fistula | 1. Concerned and anxious that more treatment may be required in the future to treat the fistula. |
| Impact of fistula activity on work | 1. Fistula discharge and pain affects ability to work and can have an impact on choice of work. 2. Need to change profession due to fistula symptoms. 3. Impact of fistula activity on concentration. |
| Sleep disturbance | 1. Sleep affected by fistula draining and fistula tract pain. |
| Restriction to sitting | 1. Fistula activity affects the ability to sit for a long period of time. 2. Fistula activity makes sitting uncomfortable. |
| Seton discomfort | 1. Pain and discomfort from seton used to treat fistula. |
| Need for a seton | 1. A seton for the fistula following surgery to repair fistula suggests that the surgery for fistula repair has failed. |
| Number of surgical attempts required to repair fistula before successful closure of fistula tract | 1. The number of planned procedures that might be needed to achieve a healed fistula. |
| Development of musculoskeletal pain | 1. Back pain and hip pain as a result of sleeping in a certain position to avoid the pressure on the fistula or the wound post surgery. |
| Limitations to movement | 1. Pain preventing exercise and restricting activity. |
| Impact of fistula activity on intimacy and sex | 1. Fistula activity affects ability to be able to have sex or be intimate, |

*verbatim report from patients

**Supplementary Table 5: Outcomes extracted from systematic review mapped to a broad outcome**

| Outcome theme | Outcome reported in study | Outcome measurement tool |
| --- | --- | --- |
| Outcomes in surgical intervention | | |
| Fistula healing | 1. No persistent fistula on clinical examination or examination under anaesthetic. 2. Closure of fistulous openings. 3. Complete closure of the fistula by the first follow-up (approximately 3 months postoperatively) without the need for additional operations. |  |
| No persistent fistula on imaging | 1. No persistent fistula on imaging (MRI, endosonography, pouchography, water   soluble contrast study). |  |
| Radiological assessment of fistula activity or healing. | 1. Assessment of fistula activity on MRI including tract size, additional tracts and change in the number of internal openings. |  |
| No collection or abscess on imaging | 1. No collection on MRI. |  |
| Volume of fistula drainage | 1. Drainage from fistula tract reported by patient. 2. Persistent drainage through fistula tract consistent with preoperative symptoms. 3. Healing of the tract defined as absence of drainage. 4. Asymptomatic patient. 5. The state of the fistula was assessed as clinically healed with the absence of discharge from the fistula tract and with evidence of closure of the fistulous openings. 6. Reduction in drainage. 7. Absence of vaginal secretion. 8. Patient reported absence of air trapping or vaginal secretion in pouch vaginal fistula. |  |
| Pain related to fistula tract | 1. Pain and inflammation of fistula. |  |
| Appearance of a new fistula or multiple fistula tracts | 1. New fistula formation following surgery. |  |
| Development of a new abscess | 1. No new septic complications at least 6 months following Redo pouch. |  |
| Recurrence of abscess or inflammation | 1. Recurrence of sepsis related to fistula tract. 2. The development of perianal abscess in the same quadrant considered as a failure of treatment. |  |
| Recurrence or persistence of a fistula | 1. No recurrence of fistula. 2. Recrudescence was defined as drainage through the same external opening of the fistula. 3. Fistula recurrence following closure of ileostomy. |  |
| A change in the location of the fistula | 1. A change in the location of the fistula. |  |
| Fistula free time | 1. Time between fistula healing and the appearance of a new fistula or recurrence of the previous fistula. |  |
| Unplanned surgery | 1. Need for intervention for abscess soon after recent intervention. 2. No additional intervention required within a period of time to achieve fistula closure. |  |
| Pouch frequency | 1. Frequency of pouch defecation. 2. Day time pouch frequency. 3. Nighttime pouch frequency. 4. Pouch defecation less than 6 times a day. 5. Patient reported satisfactory pouch function. 6. Patient satisfaction with pouch function post procedure. | Oresland pouch function (OPF)  Cleveland Clinic Foundation Pelvic Pouch Questionnaire (CCFPPQ) |
| Urgency | 1. Faecal urgency. |  |
| Anal Seepage | 1. Nocturnal pouch seepage. 2. Nocturnal seepage less than twice a week. |  |
| Continence | 1. No faecal incontinence post operatively. 2. Good sphincter function. 3. Major incontinence, minor soiling, or complete continence. 4. No nocturnal incontinence of stool. | Wexner incontinence score  Vaizey incontinence score |
| Number of surgical attempts required to repair fistula before successful closure of fistula tract | 1. Number of procedures required for fistula closure. |  |
| Quality of life assessment | 1. Assessment of quality of life post intervention. | Cleveland Global Quality of Life Score (CGQL) |
| Vaginal stenosis on clinical examination | 1. Vaginal stenosis. |  |
| Pain related to surgical wound | 1. Post operative wound pain. |  |
| Wound healing | 1. Wound healing following gracilis transposition. 2. Wound healing following fistula repair. 3. Wound infection. |  |
| Unplanned need for formation of a stoma following intervention | 1. The unplanned need for a stoma following surgical intervention due to a complication. 2. Stoma closed and gastrointestinal continuity restored without complications. |  |
| Pouch excision | 1. Pouch removal. 2. Permanent formation of end or loop ileostomy with the pouch left in situ. 3. Pouch excision. 4. Ileal pouch excision or indefinite diversion (greater than 6 months) at any time during the follow-up period for a persistent fistula was classed as failure. 5. Need for Redo IPAA or neo-pouch following pouch excision classed as pouch failure. |  |
| Procedure specific outcomes  (Excluded) | 1. No signs of anastomotic leak post. Redo IPAA. 2. Passage of fistula plug. 3. Dislodgment of fistula plug. 4. Pelvic sepsis following stoma reversal. |  |
| *Outcomes in medical intervention* | | |
| Endoscopic mucosal healing | 1. Mucosal healing 2. Endoscopic remission 3. Remission of pouchitis | Luminal disease activity index |
| Remission of fistula symptoms (Excluded as duplication) | 1. Improvement in fistula drainage. 2. Complete clinical response was defined as resolution of symptoms. Partial clinical response was defined as improvement in symptoms. 3. Cessation of fistula drainage is complete response. 4. Reduction in fistula drainage is partial response. 5. Persistent fistula drainage is no response. 6. The primary outcome was achievement of either clinical response or clinical remission at week 14. |  |
| Abdominal pain  (Excluded as outcome not related to management of fistula) | 1. Improvement in abdominal pain following biologics |  |
| Adverse effect of medications used to treat fistula | 1. Myalgia and malaise related to Anti-TNF infusion. 2. Safety outcomes include need for subsequent antibiotics, serious infection while on Anti-TNF. |  |
| Measurement of CRP | 1. Reduction in CRP following biologics. |  |
| Luminal disease activity index | 1. Change in validated scores with biologics. | Pouchitis Disease Activity Index (PouchitisDAI)  modified Pouchitis disease activity index (mPDAI)  Perianal Disease Activity Index (PDAI)  Harvey Bradshaw Index  Simple Clinical Colitis Activity Index |
| Global quality of life assessment | 1. Quality of life assessment using tools for patients with inflammatory bowel disease or ileoanal pouch (not validated for patients with a fistula) | Inflammatory Bowel Disease Questionnaire (IBDQ)  Short Form 36 (SF-36) |

**Supplementary Table 6: Outcomes included in the Delphi exercise with lay definitions**

| Source | Outcome | Lay definition | COMET domain |
| --- | --- | --- | --- |
| Both | **Pouch frequency** | The number of times the pouch needs to emptied following treatment of a fistula | **Gastrointestinal outcome** |
| Both | **Anal Seepage** | The experience of slow leakage of pouch contents (stool/mucus) through the anus following treatment of a fistula. | **Gastrointestinal outcome** |
| Both | **Continence** | Being able to control the pouch contents (stool) using the muscles of the anus following treatment of a fistula. | **Gastrointestinal outcome** |
| Both | **Urgency** | The feeling of sudden need to empty the pouch and being unable to delay this following treatment of a fistula. | **Gastrointestinal outcome** |
| Both | **Pain related to fistula tract** | Pain that is only related to fistula openings or the fistula tract. | **Gastrointestinal outcome** |
| Both | **Volume of fistula discharge** | The amount of discharge of any type (pus/stool/blood/mucus) from fistula tract following treatment. | **Gastrointestinal outcome** |
| Both | **Development of a new abscess** | Formation of a new abscess (collection of pus or infection) including multiple abscesses. | **Infection and infestation outcomes** |
| Both | **Recurrence of abscess or inflammation** | Abscess or inflammation that reappears following surgery or antibiotics to treat it. | **Infection and infestation outcomes** |
| Both | **Appearance of a new fistula or multiple fistula tracts** | A new fistula appears somewhere else around the bottom area (perineum/anus/vagina), or several new fistula develop following intervention. | **Gastrointestinal outcome** |
| Both | **Recurrence or persistence of a fistula** | Fistula persists or reappears following a period of healing after surgery or medical treatment. | **Gastrointestinal outcome** |
| Both | **Fistula healing** | This describes a fistula that has healed. | **Gastrointestinal outcome** |
| Both | **Pain related to surgical wound** | This is the severity of pain from the wounds from fistula surgery. This includes pain related to dressings or wound packing. | **Gastrointestinal outcome** |
| Both | **Unplanned surgery** | The need for unplanned surgery as an emergency soon after an operation to repair fistula or manage abscess. | **Need for further intervention** |
| Both | **Unplanned need for formation of a stoma following intervention** | The unplanned need to have a stoma following surgery to repair a fistula. | **Need for further intervention** |
| Both | **Pouch excision** | The need to have an operation to remove the pouch following surgery to repair a fistula. | **Need for further intervention** |
| Both | **Wound healing** | how quickly the surgical wounds heal following surgery for a fistula. | **Gastrointestinal outcome** |
| Both | **Number of surgical attempts required to repair fistula before successful closure of fistula tract** | The number of surgical procedures a patient has required before the fistula eventually healed. | **Need for further intervention** |
| Patient interviews | **Local skin irritation** | Local skin irritation around fistula openings due to discharge. | **Gastrointestinal outcome** |
| Patient interviews | **Pain related to an abscess** | Any pain that comes from an abscess (collection of pus or infection). | **Gastrointestinal outcome** |
| Patient interviews | **Anal pain** | General pain or discomfort from the anus | **Gastrointestinal outcome** |
| Patient interviews | **Feeling unwell or malaise** | Feeling ill or sick because of the fistula. | **Gastrointestinal outcome** |
| Patient interviews | **Seton discomfort** | A measure of how comfortable or painful a seton is (chaffing/irritation associated with a seton). | **Gastrointestinal outcome** |
| Patient interviews | **Need for a seton** | The need to have a seton to treat or manage symptoms of fistula. | **Need for further intervention** |
| Patient interviews | **Limitations to movement** | Restriction of movement such as walking, bending, running, cycling, swimming because of fistula symptoms. | **Physical functioning** |
| Patient interviews | **Development of musculoskeletal pain** | Joint and muscle ache as a result of trying to get into a comfortable position with the fistula. | **Physical functioning** |
| Patient interviews | **Restriction to sitting** | Fistula symptoms that restrict prolonged periods of sitting or require the use of extra cushions/ring cushion. | **Physical functioning** |
| Patient interviews | **Pad usage** | The need to use pads for fistula discharge. | **Physical functioning** |
| Patient interviews | **Sleep disturbance** | Impact of fistula symptoms and fistula activity on quality of sleep. | **Physical functioning** |
| Patient interviews | **Impact of fistula activity on intimacy and sex** | Unable to have sex or be physically close to someone because of fistula symptoms. | **Physical functioning, emotional functioning or well-being and social functioning** |
| Patient interviews | **Impact of fistula activity on work** | Fistula symptoms affect the ability to work or complete daily activities/chores. | **Role functioning** |
| Patient interviews | **Psychological impact of needing treatment for fistula** | Worry or anxiety about the need for further treatment for fistula. | **Emotional functioning or well-being** |
| Patient interviews | **Worried that exercise or sex might worsen symptoms** | Worried that sex or exercise might worsen symptoms of the fistula. | **Emotional functioning or well-being and social functioning** |
| Patient interviews | **Body image** | Positive or negative perception of self. | **Emotional functioning or well-being** |
| Patient interviews | **Fear and anxiety related to impact on pouch function** | Worried about losing the pouch or the impact of fistula surgery on pouch function (including incontinence or requiring further treatment with a stoma for the fistula). | **Emotional functioning or well-being** |
| Patient interviews | **Fatigue** | Feeling weak or tired. | **General outcome** |
| Systematic review | **A change in the location of fistula opening** | Surgery that changes the location of where the fistula discharges from; for example, an anal fistula that becomes a vaginal fistula following intervention. | **Gastrointestinal outcome** |
| Systematic review | **No collection or abscess on imaging** | Using radiological imaging (such as MRI or ultrasound) to assess whether there is an abscess or collection of pus following treatment. | **Gastrointestinal outcome** |
| Systematic review | **Radiological assessment of fistula activity or healing** | Using radiological imaging (such as MRI or ultrasound) to assess whether the fistula has worsened, improved, or remained the same. | **Gastrointestinal outcome** |
| Systematic review | **Endoscopic mucosal healing** | In patients with a fistula who are receiving medical treatment for inflammatory bowel disease such as Crohn’s disease or pouchitis endoscopy may be used to check bowel wall for healing or response to treatment. | **Gastrointestinal outcome** |
| Systematic review | **Fistula free time** | This is the time (days/months/years) period between a healed fistula and a recurrence, or development of a new fistula. | **Gastrointestinal outcome** |
| Systematic review | **Vaginal stenosis on clinical examination** | Vaginal canal may become narrowed because of fistula activity, inflammation, and surgery to repair fistula. | **Gastrointestinal outcome** |
| Systematic review | **Adverse effect of medications used to treat fistula** | The side effects of medications used to treat a fistula in patients. | **Adverse events** |
| Systematic review | **Measurement of CRP** | C-reactive protein (CRP) is a marker of inflammation which can be measured using a blood test. This can be measured in patients with inflammatory bowel disease such as Crohn’s disease. | **Infection and infestation outcomes** |
| Systematic review | **Luminal disease activity index** | Clinicians may use validated scores to measure inflammation in the bowel in inflammatory bowel disease. These scores may be useful in measuring response to medical treatment used to treat fistula in patients with inflammatory bowel disease such as Crohn’s disease. | **Gastrointestinal outcome** |
| Systematic review | **No persistent fistula on imaging** | Using radiological imaging (such as MRI or ultrasound) to assess whether there is still a fistula following treatment. | **Gastrointestinal outcome** |
| Systematic review + SMG | **Global quality of life assessment** | A validated measurement tool to assess the quality of life of patients with a pouch fistula. | **Global quality of life** |

**Supplementary Table 7: Delphi participant demographics**

|  | **Round 1** | | **Round 2** | |
| --- | --- | --- | --- | --- |
| **Clinicians** | **N= 41** | | **N=38** | |
|  | Surgeons, Gastroenterologists, Radiologists  N=35 (%) | Clinical nurse specialist  N=6 (%) | Surgeons, Gastroenterologists, Radiologists  N=33 (%) | Clinical nurse specialists  N = 5 (%) |
| Male | 26 (74) |  | 25 (76) |  |
| Female | 9 (26) | 6 (100) | 8 (24) | 5 |
| **Years of practice** | | | | |
| 0-5 | 15 (43) | 2 (33) | 15 (46) | 2 (40) |
| 6-10 | 6 (17) | 1 (17) | 5 (15) |  |
| 11-20 | 10 (29) | 3 (50) | 9 (27) | 3 (60) |
| >20 | 4 (11) |  | 4 (12) |  |
| **Subspeciality** | | | | |
| Colorectal surgeon with a specialist interest ileoanal pouch | 11 (31) |  | 11 (33) |  |
| General colorectal surgeon | 9 (26) |  | 8 (24) |  |
| Proctologist | 2 (6) |  | 2 (6) |  |
| Gastroenterologist with a specialist interest in ileoanal pouch | 6 (17) |  | 6 (18) |  |
| General gastroenterologist | 2 (6) |  | 2 (6) |  |
| Specialist GI radiologist | 5 (100) |  | 4 (12) |  |
| General radiologist | 0 |  | 0 |  |
| **No. of patients seen annually** | | | | |
| 0-5 | 19 (54) | 3 (50) | 18 (55) | 2 (40) |
| 6-10 | 9 (26) | 3 (50) | 9 (27) | 3 (60) |
| 11-15 | 2 (6) |  | 2 (6) |  |
| >15 | 5 (14) |  | 4 (12) |  |
| **Work setting** | | | | |
| District General Hospital | 1(3) |  | 1 (3) | 0 |
| Tertiary referral centre | 33 (94) | 6 (100) | 32 (97) | 5 (100) |
| Private hospital | 1(3) |  | 0 | 0 |
| **Experience** | | | | |
| Clinician | 22 (63) | 6 (100) | 20 (61) | 6 (100) |
| Researcher | 4 (11) |  | 4 (12) |  |
| Both | 9 (26) |  | 9 (27) |  |
| **Patients** | **N=37** | | **N= 36** | |
| Male | 14 (38) |  | 13 (36) |  |
| Female | 23 (62) |  | 23 (64) |  |
| **Age** | | | | |
| 18-20 |  |  |  |  |
| 21-30 | 1 (3) |  | 1 (3) |  |
| 31-40 | 8 (21) |  | 7 (21) |  |
| 41-50 | 12 (32) |  | 12 (32) |  |
| 51-60 | 11 (30) |  | 11 (30) |  |
| 61-70 | 4 (11) |  | 4 (11) |  |
| 71-80 | 1 (3) |  | 1 (3) |  |
| **Ethnicity** | | | | |
| White/Caucasian | 29 (78) |  | 28 (78) |  |
| Other | 1 (3) |  | 1 (3) |  |
| Not stated | 7 (19) |  | 7 (19) |  |
| **Sexuality** | | | | |
| Heterosexual | 28 (75) |  | 27(75) |  |
| Other | 1 (3) |  | 1 (3) |  |
| Not stated | 8 (22) |  | 8 (22) |  |
| **Type of fistula** | | | | |
| Pouch anal | 18 (50) |  | 17 (47) |  |
| Pouch vaginal | 10 (28) |  | 10 (28) |  |
| Both | 8 (22) |  | 8 (22) |  |
| **Duration of fistula** | | | | |
| 0-6 months |  |  |  |  |
| 7-12 months | 1 (3) |  | 1 (3) |  |
| >12 months | 70 (69) |  | 25 (69) |  |
| I do not have a fistula | 10 (27) |  | 10 (28) |  |
| **Current fistula status** | | | | |
| Current symptomatic fistula, not had treatment yet | 2 (6) |  | 2 (6) |  |
| Previously had a fistula that has been treated and healed, or given me no symptoms | 11 (30) |  | 11 (30) |  |
| Undergone treatment but fistula still active | 22(58) |  | 21 (58) |  |
| Other | 2 (6) |  | 2 (6) |  |
| **Hospital setting for treatment (select all that apply)** | | | | |
| DGH | 16 (43) |  | 16 (43) |  |
| Tertiary referral centre | 21 (57) |  | 20 (57) |  |
| Private Hospital | 0 |  | 0 |  |
| **All participants** | | | | |
| **Country of residence** | **N= 77** | |  | |
| UK | 62 (79) | | 58 (79) | |
| Italy | 3 (4) | | 3 (4) | |
| USA | 2 (3) | | 2 (3) | |
| Australia | 2 (3) | | 2 (3) | |
| Belgium | 2 (3) | | 2 (3) | |
| Turkey | 2 (3) | | 2 (3) | |
| Germany | 1 (1) | | 1 (1) | |
| Poland | 1 (1) | | 1 (1) | |
| Sweden | 1 (1) | | 1 (1) | |
| China | 1 (1) | | 1 (1) | |
| Malaysia | 1 (1) | | 1 (1) | |

**Supplementary Table 8: Consensus meeting voting results**

| Outcome | Vote 1 | | Vote 2 | | Consensus |
| --- | --- | --- | --- | --- | --- |
|  | Yes | No | Yes | No |  |
| A change in the location of fistula opening | 43% | 57%** |  |  | OUT |
| Need for a seton | 43% | 57%** |  |  | OUT |
| Worried that exercise or sex might worsen symptoms | 50% | 50% |  | 100% | OUT |
| Body image | 64% | 36%* |  |  | OUT |
| Fatigue | 46% | 54% |  |  | OUT |
| No collection or abscess on imaging | 93% | 7% |  |  | IN |
| Radiological assessment of fistula activity or healing | 93% | 7% |  |  | IN |
| Anal Seepage | 71% | 29% |  |  | IN |
| Continence | 100% |  |  |  | IN |
| Urgency | 87% | 13% |  |  | IN |
| Pain related to fistula tract | 100% |  |  |  | IN |
| Pain related to an abscess | 80% | 20% |  |  | IN |
| Volume of fistula discharge | 80% | 20% |  |  | IN |
| Development of a new abscess | 100% |  |  |  | IN |
| Recurrence of abscess or inflammation | 100% |  |  |  | IN |
| Appearance of a new fistula or multiple fistula tracts | 80% | 20% |  |  | IN |
| Recurrence or persistence of a fistula | 100% |  |  |  | IN |
| Fistula healing | 93% | 7% |  |  | IN |
| Need for unplanned surgery following intervention | 87% | 13% |  |  | IN |
| Unplanned need for formation of a stoma following intervention | 93% | 7% |  |  | IN |
| Pouch excision | 100% |  |  |  | IN |
| Sleep disturbance | 93% | 7% |  |  | IN |
| Impact of fistula activity on intimacy and sex (including body image) | 100% |  |  |  | IN |
| Impact of fistula activity on work | 79% | 21% |  |  | IN |
| Exercise and limitations to movement | 100% |  |  |  | IN |
| Global quality of life assessment | 100% |  |  |  | IN |
| Pad usage | 64% | 36% |  |  | OUT |
| Wound healing (not fistula healing) | 60% | 40% |  |  | OUT |
| Number of surgical attempts required to repair fistula before successful closure of fistula tract | 36% | 64% |  |  | OUT |
| Anal pain | 53% | 47% |  |  | OUT |
| Feeling unwell or malaise | 8% | 92% |  |  | OUT |
| Seton discomfort | 29% | 71% |  |  | OUT |
| Psychological impact of needing treatment for fistula | 57% | 43%* |  |  | OUT |
| Fear and anxiety related to impact on pouch function | 21% | 79% |  |  | OUT |
| Fistula free time | 29% | 71% |  |  | OUT |
| Vaginal stenosis on clinical examination following intervention | 55% | 45% |  |  | OUT |
| *Combined outcomes* |  |  |  |  |  |
| Global quality of life assessment | 87% | 13% |  |  | Include |
| Pain related to fistula or surrounding area | 92% | 8% |  |  | Include |
| Impact on quality of life of discharge | 100% |  |  |  | Include |
| Fistula healing (clinical and radiological)  (measured over a period of time) | 79% | 21% |  |  | Include |
| Need for rescue intervention (minor and major) | 100% |  |  |  | Include |
| New fistula or abscess | 57% | 43%** |  |  | Included by SMG |

* Not discarded, combined with other outcome

**>70% patients voted to discard

**Supplementary Figure 1: PRISMA for systematic review of outcomes in the management of pouch anal and vaginal fistula**

References from other sources **(n = 6 )**

Citation searching (n = 5)

Studies from databases/registers **(n = 1962)**

**Identification**

References removed **(n = 173)**

Duplicates identified by Covidence (n = 173)

Studies screened **(n = 1794)**

Studies excluded **(n = 1610)**

Studies sought for retrieval **(n = 184)**

Studies not retrieved **(n = 1)**

**Screening**

Studies assessed for eligibility **(n = 183)**

Studies excluded **(n = 113)**

Duplicate (n = 23)

Wrong study design (n = 46)

Not English language (n = 11)

Abstract only (n =33)

**Included**

Studies included in review **(n =70)**
