# Supplementary material for: PAVFCOS: The development of a core outcome set for pouch anal and vaginal fistula
Source: Colorectal Dis. 2025 Aug 8;27(8):e70184. doi: 10.1111/codi.70184 (PMC12334349; doi:10.1111/codi.70184)
Supplement: Supplementary file 2 — Data S1. [file CODI-27-e70184-s001.docx]

**The development of a Core Outcome Set for pouch anal and vaginal fistula using a Delphi study**

The following is a guide for conducting patient interviews to determine patient reported outcomes in the management of pouch anal and pouch vaginal fistulae.

**Screening and recruitment of patient participants**

Outpatient colorectal clinics and a prospectively maintained fistula database will be screened for patients with pouch anal or pouch vaginal fistulae. A purposive approach to sampling will be used to ensure demographic diversity and a wide representation of complexity in fistula characteristics within the study population. We have established certain criteria to ensure representation of contrasting perspectives and experiences of pouch anal and pouch vaginal fistulae. These criteria will be used to generate a sampling matrix.

**Sampling matrix**

Key criteria to identify difference between participants:

1. Age at diagnosis

2. Gender

3. Ethnicity

4. Duration of disease

5. Fistula characteristics:

- Pouch anal fistula

- Pouch vaginal fistula

- Fistula Classification: Anastomotic related, Crohn’s related, Non -Crohns Inflammatory related, cryptoglandular disease related and malignancy related

- Number of fistulas

- Stage of disease: active/ remission/ persistence/ recurrence

**Fistula activity**

Mild grumbling

Moderate

Severe

Healed

6. Fistula treatment

- Number of previous surgical interventions

- Biological treatment

- Pouch excision

- Defunctioning ileostomy

**Patient interview**

The patient interviews will be used to explore patient experiences of living with a pouch anal or pouch vaginal fistula. We intend to better understand patient perspectives of what constitutes a successful outcome of fistula treatment. A semi- structured interview format will be used. This will ensure that the discussion is patient-led with occasional prompts from a pre-prepared topic guide in order to cover all key areas. The topic guide will most likely evolve with subsequent patient interviews as participants may raise important points of discussion not covered by the topic guide.

**Interview guide**

Participant number:

Date of birth:

Gender:

Ethnicity:

Age at pouch creation:

Type of fistula:

Pouch anal

Pouch vaginal

Both

Fistula stage:

active/ remission/ persistence/ recurrence

Number of fistulas:

Pouch function:

1. Introduction and consent

1. Outline the purpose of the study
2. Ensure patient still willing to take part
3. Check if participant agrees that the interview be audio-recorded, stored and used for presenting research findings anonymously.
4. Check if there are any questions regarding the interview procedure
5. Ask participant to complete the informed consent form

2. Explore patient experience of living with a pouch fistula

Ask about the experience of having a pouch anal or pouch vaginal fistula

- 1. ‘I understand you have (had) a pouch anal/ pouch vaginal fistula. Can you tell me more about that?’
  2. ‘How soon after your pouch was created did you notice symptoms which then turned out to be from a fistula?’/‘Could you tell me about how you first found out you had a fistula?’
  3. ‘What type of symptoms did you have after being diagnosed with a fistula’/’ Did your pouch function deteriorate at the same time as the fistula developed?’
  4. ‘Can you tell me what life with a fistula is like for you?’/’What is the impact on daily life?’/’Did it impact on your social life?’/’Did it impact on your sex life?’/’ Does it impact your work life?’
  5. ‘What do you feel is the worst aspect of having a fistula?’
  6. ‘How do you manage the fistula?’

3. Ask about the treatment that was offered and how they decided about undergoing treatment

1. ‘What treatment(s) were you offered, and which did you choose/ receive?’
2. ‘Were you aware of the pros and cons of the different treatment options?’
3. What made you decide on one treatment over the other?’/’What factors did you consider in deciding on a treatment?’

4. Ask about the effects that treatment had/is having

- 1. ‘Was your treatment successful, and why?’/’How did the treatment impact on daily life/ working life/ social life/sex life?’/’How did you notice that the fistula treatment worked (if it worked)?’/ ‘What factors are important to you in deciding whether a treatment has worked?’
  2. ‘Did you experience any negative effects right after treatment or later on?’
  3. ‘How bad were these negative effects? Would you choose this treatment again or recommend it to a family member?’
  4. ‘What do you feel is the worst aspect of the treatment?’
  5. **‘What would you say is the most important result after the treatment?’**
  6. ‘Did your perspective on what is important change over time?’
  7. ‘Overall, were you satisfied with the results of the treatment? Did the treatment match your expectations?’

5. Ask about concerns for the future

- 1. **‘Do you have any concerns for the future? What is it that you are most concerned about? Has this changed over time?’**

6. Outcomes believed to be relevant and important to include in trials and why

1. Ask about important results of treatment
2. In future, we want to be able to select a treatment that best suits the patient’s preferences. Based on your experience, ‘what would be the most important outcome/result of the treatment?’

7. Closing

1. Ask whether there is anything else they would like to talk about
2. Check if there are any questions
